# Supplementary material for: Preserved extrastriate visual network in a monkey with substantial, naturally occurring damage to primary visual cortex
Source: eLife. 2019 May 23;8:e42325. doi: 10.7554/eLife.42325 (PMC6533062; doi:10.7554/eLife.42325)
Supplement: Transparent reporting form [file elife-42325-transrepform.docx]

***eLife’s* transparent reporting form**

We encourage authors to provide detailed information *within their submission* to facilitate the interpretation and replication of experiments. Authors can upload supporting documentation to indicate the use of appropriate reporting guidelines for health-related research (see [EQUATOR Network](http://www.equator-network.org/%20)), life science research (see the [BioSharing Information Resource](https://biosharing.org/)), or the [ARRIVE guidelines](http://www.plosbiology.org/article/info:doi/10.1371/journal.pbio.1000412) for reporting work involving animal research. Where applicable, authors should refer to any relevant reporting standards documents in this form.

If you have any questions, please consult our Journal Policies and/or contact us: [editorial@elifesciences.org](mailto:editorial@elifesciences.org).

**Sample-size estimation**

- You should state whether an appropriate sample size was computed when the study was being designed
- You should state the statistical method of sample size computation and any required assumptions
- If no explicit power analysis was used, you should describe how you decided what sample (replicate) size (number) to use

Please outline where this information can be found within the submission (e.g., sections or figure legends), or explain why this information doesn’t apply to your submission:

As this is a rare case study of a Rhesus macaque likely born without primary visual cortex, we could not undertake sample size estimation. We compared structural and functional MRI data of this special case to the same measures from groups of animals of up to 10. In order to maximize control groups, we also utilized previously obtained data for the anatomical analysis. Details on animal numbers and inclusion criteria for different parts of the study are detailed in the Methods, especially the sections on ‘Animals’ and ‘Analysis of functional MRI data’. Each section in the Results states the number of animals and inclusion criteria; where appropriate, figures show and compare individual results across all included animals (Figures 1, 3, 4, 5, 6, 8, 9, 10, 11).

**Replicates**

- You should report how often each experiment was performed
- You should include a definition of biological versus technical replication
- The data obtained should be provided and sufficient information should be provided to indicate the number of independent biological and/or technical replicates
- If you encountered any outliers, you should describe how these were handled
- Criteria for exclusion/inclusion of data should be clearly stated
- High-throughput sequence data should be uploaded before submission, with a private link for reviewers provided (these are available from both GEO and ArrayExpress)

Please outline where this information can be found within the submission (e.g., sections or figure legends), or explain why this information doesn’t apply to your submission:

Each data point pertains to results from one hemisphere from an individual animal. Details on animal numbers and inclusion criteria for different parts of the study are detailed in the Methods, especially the sections on ‘Animals’ and ‘Analysis of functional MRI data’. Each section in the Results states the number of animals and inclusion criteria. For functional MRI analysis, we included only scan sessions (and animals) that showed significant activation in the LGN (z-statistic > 2.3) in response to the checkerboard stimulus – to control for the effect of general anaesthesia on the CNS. For anatomical analysis, we included all available data. Figures generally show individual results and compare all included individual results across the cohort (Figures 1, 3, 4, 5, 7, 8, 9, 10, 11) and where appropriate between hemispheres.

**Statistical reporting**

- Statistical analysis methods should be described and justified
- Raw data should be presented in figures whenever informative to do so (typically when N per group is less than 10)
- For each experiment, you should identify the statistical tests used, exact values of N, definitions of center, methods of multiple test correction, and dispersion and precision measures (e.g., mean, median, SD, SEM, confidence intervals; and, for the major substantive results, a measure of effect size (e.g., Pearson's r, Cohen's d)
- Report exact p-values wherever possible alongside the summary statistics and 95% confidence intervals. These should be reported for all key questions and not only when the p-value is less than 0.05.

Please outline where this information can be found within the submission (e.g., sections or figure legends), or explain why this information doesn’t apply to your submission:

We provide and plot individual animals’ or hemispheres’ results for each included animal (or each hemisphere), see Figures 1, 3, 4, 5, 6, 8, 9, 10, 11. Where we provide summaries we give the mean and the SD (Results section: ‘Subcortical visual areas are structurally normal in monkey S’) or the median and the 95% confidence interval (Figure 10) of the population, as appropriate.

(For large datasets, or papers with a very large number of statistical tests, you may upload a single table file with tests, Ns, etc., with reference to sections in the manuscript.)

**Group allocation**

- Indicate how samples were allocated into experimental groups (in the case of clinical studies, please specify allocation to treatment method); if randomization was used, please also state if restricted randomization was applied
- Indicate if masking was used during group allocation, data collection and/or data analysis

Please outline where this information can be found within the submission (e.g., sections or figure legends), or explain why this information doesn’t apply to your submission:

This is a unique animal case study with a gross brain abnormality compared to typical controls. There is no element of assigning experimental groups. However, all the behavioural data described was obtained before the animal’s brain abnormality had been discovered, so it has been collected ‘blind’.

**Additional data files (“source data”)**

- We encourage you to upload relevant additional data files, such as numerical data that are represented as a graph in a figure, or as a summary table
- Where provided, these should be in the most useful format, and they can be uploaded as “Source data” files linked to a main figure or table
- Include model definition files including the full list of parameters used
- Include code used for data analysis (e.g., R, MatLab)
- Avoid stating that data files are “available upon request”

Please indicate the figures or tables for which source data files have been provided:

As we plot or show individual results, we have not made separately available source files for the graphs. We provide in the supplementary material (Figure 4 – figure supplement 2) a table of anaesthetic parameters linked to weight and outcome of the individual functional MRI scanning sessions collected for this study. We make the central source structural and functional MRI data available online through the GIN data repository at <https://web.gin.g-node.org/hbridge_oxford/brainwithoutv1>.
